# Psychedelics for the Treatment of Obsessive–Compulsive Disorder: Efficacy and Proposed Mechanisms

**DOI:** 10.1093/ijnp/pyae057

**Published:** 2024-11-29

**Authors:** Helen M Collins

**Affiliations:** MRC Brain Network Dynamics Unit, Nuffield Department of Clinical Neurosciences, University of Oxford, Oxford, UK

**Keywords:** obsessive–compulsive disorder, psychedelics, psilocybin

## Abstract

Psychedelics are emerging as potential treatments for a range of mental health conditions, including anxiety and depression, treatment-resistant depression, and substance use disorders. Recent studies have also suggested that the psychedelic psilocybin may be able to treat obsessive–compulsive disorder (OCD). Since the 1960s, case studies have reported improvements to obsessive and compulsive behaviors in patients taking psychedelics recreationally. The effects of psilocybin were then systematically assessed in a small, open-label trial in 2006, which found that psilocybin significantly reduced the symptoms of OCD. Reduced compulsive behaviors have also been seen in rodent models of OCD after administration of psilocybin. Nonetheless, the mechanisms underlying the effects of psychedelics for OCD are unclear, with hypotheses including their acute pharmacological effects, changes in neuroplasticity and resting state neural networks, and their psychological effects. This review will evaluate the evidence supporting the theory that psychedelics can be used for the treatment of OCD, as well as the data regarding claims about their mechanisms. It will also discuss issues with the current evidence and the ongoing trials of psilocybin that aim to address these knowledge gaps.

## BACKGROUND

Obsessive–compulsive disorder (OCD) is a chronic psychological disorder characterized by disturbing intrusive and persistent thoughts, and/or repetitive behaviors or compulsions (Stein et al., 2019), with a lifetime prevalence of 1.5%-3% (based on adults in the United States) (Robins et al., 1984; Karno et al., 1988; Kessler et al., 2012). OCD is a highly heterogeneous disorder, with symptoms varying greatly between patients (Van Schalkwyk et al., 2016); for instance, different subpopulations exhibit different dimensions of obsessive–compulsive behaviors (eg, checking, washing, repeating, counting, ordering) (Belayachi and Van der Linden, 2017) and have different motivations for these behaviors (eg, harm avoidance, incompleteness, intolerance of uncertainty) (Bragdon and Coles, 2017).

Despite its pervasiveness, there are few effective treatments for OCD. Selective serotonin (5-hydroxytryptamine, 5-HT) reuptake inhibitors (SSRIs) are the first-line pharmacological treatments for OCD (Jenike, 1993; El Mansari and Blier, 2006; Walker et al., 2015), but their therapeutic effects can take several weeks to emerge (Fineberg et al., 1992; Montgomery and Manceaux, 1992; Issari et al., 2016; Swierkosz-Lenart et al., 2023). Moreover, 30%-60% of patients do not respond to SSRI treatment and are considered treatment-resistant (McDougle et al., 2000; Denys et al., 2003; Denys, 2006; Fineberg and Robbins, 2021). Alternative pharmacotherapies include lithium, buspirone, and pindolol, but their efficacies are similarly low (Bergqvist et al., 1999b). Cognitive behavioral therapy and exposure and response prevention therapy are psychological treatments for OCD; however, these approaches require highly trained specialists and regular sessions to be effective, limiting their availability (Ching et al., 2023). More invasive approaches include surgical ablation of parts of the cerebral cortex, deep brain stimulation, and transcranial magnetic stimulation (Ehrmann et al., 2022; Buot et al., 2023), but these therapies can have extensive side effects (Khan et al., 2023). Innovative therapeutic approaches, especially for treatment-resistant OCD, are therefore needed to improve the lives of patients with OCD.

After decades of prohibition of their clinical and scientific use, one increasingly used approach in modern psychiatry is the use of psychedelics to treat mental illness. Classical psychedelics include psilocybin, lysergic acid diethylamide (LSD), mescaline, and *N,N*-dimethyltryptamine (Sousa et al., 2022). Psilocybin (active ingredient psilocin) is a naturally occurring indolealkylamine derived from *Psilocybe* mushrooms (Hofmann et al., 1959). Psilocybin has a complex pharmacological profile, acting as an agonist at 5-HT_2A_, 5-HT_2C_, and 5-HT_1A_ receptors (Delgado and Moreno, 1998b; Aghajanian and Marek, 1999; Passie et al., 2002; Halberstadt and Geyer, 2011; Pokorny et al., 2016). Similarly, LSD is an agonist of several 5-HT receptors, including 5-HT_2A/2C_ and 5-HT_1A_ (Passie et al., 2008). Classical psychedelics produce acute psychological effects, colloquially referred to as “peaks” or “trips,” which include altered visuospatial, motion, and time perception, hallucinations, changes in information processing, and profound changes in mood (Studerus et al., 2011). They can also produce feelings of meaningfulness, insight into one’s emotions, and a feeling of unity with others (Carhart-Harris et al., 2012a; Roseman et al., 2018). The psychoactive effects of psychedelics substances are predominantly mediated by 5-HT_2A_ receptor activation, with its occupancy tightly associated with the intensity of hallucinations (Studerus et al., 2011; Roseman et al., 2018).

Ketamine is another psychoactive compound that has also been trialed for the treatment of mental health conditions (Molero et al., 2018). Ketamine produces distortions to perception, altered states of consciousness, and a feeling of disconnect from reality, as well as profound psychomimetic experiences and hallucinations at subanaesthetic doses (Krupitsky and Grinenko, 1997; Nutt, 2019). Nonetheless, it is not considered a classical psychedelic due to its distinct pharmacological mechanisms, and thus the effects of ketamine on OCD are beyond the scope of this review and can be found systematically assessed elsewhere (Bandeira et al., 2022).

Promising early data suggest that psychedelics, and particularly psilocybin, may be effective treatments for depression and anxiety (Griffiths et al., 2016; Ross et al., 2016; Evans et al., 2018; Grassi et al., 2020; Davis et al., 2021; Martinotti et al., 2021; van Amsterdam and van den Brink, 2022), mood disorders in late-stage cancer (Grob et al., 2011), treatment-resistant depression (TRD) (Carhart-Harris et al., 2016a, 2021), and substance use disorders (Moreno et al., 2006). Studies of TRD have demonstrated the safety and tolerability of psilocybin when used in a clinical setting (Carhart-Harris et al., 2016a; Majić et al., 2017; Sousa et al., 2022), with the US Food and Drug Administration now designating psilocybin as a “breakthrough therapy” for both anxiety and major depressive disorder. Many potential mechanisms have been proposed for the clinical benefits of psychedelics for mental health conditions, ranging from their pharmacological effects on brain circuitry to psychological theories of increased openness and flexibility. Nonetheless, data for specific disorders are still limited.

Given the limited efficacy of the current first-line treatments for OCD, the success of psilocybin in improving symptoms of other mental health conditions, and promising preclinical data, psychedelics could be used as alternative treatments for OCD, especially for treatment-resistant cases. This review will evaluate the clinical evidence of the effectiveness of classical psychedelics for the treatment of OCD. It will also discuss hypotheses on their mechanisms of action, as well as ongoing trials to further explore their therapeutic effects. The literature included in the following discussion was identified using a thorough, albeit not systematic, review using PubMed, Google Scholar, and Web of Science.

## CLINICAL EFFICACY OF PSYCHEDELICS FOR OCD

### Case Reports

Although psychedelic therapy currently undergoing a resurgence in research, its use dates back to the 1950s and 1960s, when approximately 4000 patients underwent psychedelic psychotherapy for various mental health conditions (Bakalar and Grinspoon, 1990; Andersen et al., 2021). Early case studies reported that patients with obsessive and/or compulsive behaviors had diminished symptoms after taking *Psilocybe* mushrooms (Savage et al., 1964; Brandrup and Vanggaard, 1977) (see Table 1 for a summary of the clinical data). Similarly, patients with diagnosed OCD who took LSD and psilocybin reported reductions in their OCD symptoms (Leonard and Rapoport, 1987; Hanes, 1996; Moreno and Delgado, 1997). More recently, 2 case reports described how patients taking approximately 2 g of Psilocybe mushrooms every 2-3 weeks saw qualitative improvements to their OCD symptoms, which lasted for several weeks after each dose (Wilcox, 2014; Lugo-Radillo and Cortes-Lopez, 2021).

**Table 1.** Summary of Published Reports on the Effects of Psychedelics in Patients With OCD.

Study	Study type	Participants	Drug	Dose, frequency, and administration	Outcome
Savage et al., 1964	Questionnaire	113 participants	LSD and mescaline	200-300 μg LSD and 200-400 μg mescaline given initially, with 300 μg LSD given later if needed, given in a clinical setting	Improved subjective quality of life, reduced compulsive habits and behaviors
Brandrup and Vangaard, 1977	Case report	30-year-old man with “compulsive-neurotic condition”	LSD	Taken repeatedly over 11.5 years	Cured of compulsive symptoms, reported favorable changes to his personality
Leonard and Rappoport, 1987	Case report	17-year-old patient with severe OCD and substance abuse	LSD and psilocybin	N/A	Total relief of OCD symptoms after taking psychedelic drugs
Hanes, 1996	Case report	27-year-old man with body dysmorphic symptoms (related to OCD in DSM-V)	Psilocybin	Ingested *Psilocybe* mushrooms on 3 occasions	No longer felt “deformed” when he saw himself in the mirror
Moreno and Delgado, 1997	Case report	34-year-old man with OCD	Psilocybin	Ingested 2 g dried *Psilocybe* mushrooms twice-weekly for 4 years	Improvement to obsessions and compulsions during acute psychedelic effects, which would last for 4-5 days, followed by a gradual return of symptoms. Maintained symptomatic remission for several months after stopping psilocybin ingestion before symptoms slowly returned
Moreno et al., 2006	Clinical trial	9 patients with treatment-resistant OCD	Psilocybin	100, 200, and 300 μg/kg over 4 weeks with a very low dose (25 μg) randomly inserted as control. Orally ingested in clinical environment with trained “sitters” for 8 hours	23-100% reduction in Y-BOCS scores 24 hours after ingestion, 88.9% reported at least 25% improvement in symptoms, 66.7% experienced a 50% reduction. Two patients reported improvements 1 week after ingestion, one in remission for 6 months. Neither dose nor intensity of subjective effects predicted magnitude of improvement
Wilcox, 2014	Case report	38-year-old man with OCD and severe anxiety since childhood	Psilocybin	Ingested approximately 2 g of *Psilocybe* mushrooms every 3 weeks	First exposure was anxiogenic and caused dissociation for several hours, but the next day intrusive thoughts were reduced. After each ingestion, had relief from symptoms for 3 weeks
Lugo-Radillo et al., 2021	Case report	Adult male with OCD, resistant to treatment with SSRIs and ketamine	Psilocybin (in combination with SSRI)	Ingested 2 g of *Psilocybe* mushrooms every 2 weeks	No hallucinogenic effects, but dissociation for 1 hour after ingestion, OCD symptoms completely disappeared and were significantly less for 2 weeks. Psilocybin reduced symptoms by 63%
Kelmendi et al., 2022	Case report	33-year-old with OCD (history of MDD and Tourette’s and panic disorder), never saw adequate relief from SSRIs or CBT, participant in trial NCT03356483	Psilocybin	Randomized to treatment with 0.25 mg/kg psilocybin, given in a relaxing room with facilitators, with follow-up sessions at 48 hours, 2 and 12 weeks	Previously seen benefits from using *Psilocybe* mushrooms, effects lasting 1-2 weeks. Here, psilocybin reduced Y-BOCS score from 24 (severe) to 0-2 (no symptoms) 48 hours after dosing, remained low even 12 weeks later. Also noted improvements to quality of life, relationships and reduced depression and anxiety-symptoms
Buot et al., 2023	Retrospective survey	174 adults with OCD, average age 29 years, 54% female	Psilocybin and LSD	Varied	Classical psychedelics were the only effective treatments for OCD. Dose of drug correlated with reduction in symptom severity
Moreton et al., 2023	Retrospective survey	312 participants reporting significant psychedelic experience	Varied	Varied	Subjective effects of psychedelics, eg, mystical experiences and psychological insight predicted self-reported reductions in obsessions and compulsions

Abbreviations: CBT, cognitive behavioral therapy; DSM-V, The Diagnostic and Statistical Manual of Mental Disorders, Fifth Edition; IV, intravenous; LSD, lysergic acid diethylamide; MDD, major depressive disorder; N/A, information not available; OCD, obsessive–compulsive disorder; SSRIs, selective serotonin reuptake inhibitors; Y-BOCS, Yale-Brown Obsessive Compulsive Scale.

Quantitatively, psilocybin has also been shown to reduce scores on the Yale-Brown Obsessive Compulsive Scale (Y-BOCS), a clinician-led 10-item assessment, where higher scores indicate more severe OCD (Goodman et al., 1989b, 1989c). In 1 case study, psilocybin reduced Y-BOCS score by 63% (Lugo-Radillo and Cortes-Lopez, 2021); in another, psilocybin reduced Y-BOCS score from 24 (severe OCD) to 0-2 (no symptoms) within 24 hours of its administration (Kelmendi et al., 2022). Importantly, such improvements were seen in patients who had failed to respond to other therapeutic approaches (Lugo-Radillo and Cortes-Lopez, 2021; Kelmendi et al., 2022), suggesting that psychedelics may be suitable for patients with treatment-resistant OCD.

Interestingly, observations from these case reports suggest that the hallucinogenic effects of psychedelics are not necessary for symptomatic improvement (Lugo-Radillo and Cortes-Lopez, 2021), although dissociative effects do appear common (Wilcox, 2014). The beneficial effects of psilocybin have also been reported after tolerance to the acute psychedelic effects has developed and can even persist for several months after discontinuation from repeated psilocybin exposure (Moreno and Delgado, 1997; Delgado and Moreno, 1998b). Thus, the clinical benefits of psilocybin for OCD can be distinguished from its psychomimetic effects and suggest they may retain their efficacy even if the patient develops tolerance to their acute effects.

These individual experiences provide preliminary evidence that psilocybin may have some therapeutic potential for OCD. Nonetheless, these case studies report retrospective, sporadic, and personal use (except for Kelmendi et al., 2022), and laboratory analysis of the compounds being taken was generally lacking. It is therefore difficult to determine the amounts of psilocybin being taken or at what frequency, or even if the mushrooms were of the *Psilocybe* variety (Wilcox, 2014; Jacobs, 2020). Data from controlled studies are therefore needed to reliably assess efficacy of psilocybin for OCD.

### Clinical Trials

To date, there has only been 1 clinical trial of the safety, tolerability, and efficacy of psilocybin for OCD. Moreno and colleagues (2006) performed a multidose, open-label study of 9 patients who had failed to respond to at least 1 standard treatment for OCD. A dose of psilocybin was paired with unstructured psychological support in a controlled environment once a week for 4 weeks; patients received a low dose (100 μg/kg), a medium dose (200 μg/kg), and then a high dose (300 μg/kg). A very low dose (25 μg/kg) was randomly inserted in a double-blind fashion into 1 session after the first dose. Patients were monitored before, during, and after psilocybin ingestion using the Y-BOCS scale and hallucinogen rating scores. Overall, a 23%-100% decrease in OCD symptoms was seen 24 hours after psilocybin ingestion; 8 patients reported a 25% reduction in symptoms, and 6 saw their symptoms reduce by 50%. These effects were seen well beyond the 2- to 3-hour half-life of psilocybin (Brown et al., 2017; Holze et al., 2023; Ley et al., 2023)—2 patients reported symptomatic improvements 1 week after ingestion, and another was in remission for several months. This study also demonstrated the safety and tolerability of psilocybin for OCD, with only mild hypertension reported as a side effect, which resolved within 6 hours (Moreno et al., 2006).

Nonetheless, the very low dose of psilocybin, which was included as a negative control dose, also reduced OCD symptoms, meaning this study was not placebo-controlled. This unexpected finding may indicate that the low dose was still biologically active. Alternatively, there may have been carry-over effects between doses and/or expectancy effects, as patients had previously experienced at least 1 dose of psilocybin and were selected for the trial based on having previously tolerated indole-based psychedelics. Given that neither the dose of psilocybin nor the intensity of its psychedelic effects were predictive of the magnitude of change in OCD scores (lack of dose-dependent effect), it is likely that at least some of the benefits of psilocybin did not arise from its pharmacological actions. The open-label nature of the trial may also have biased patients toward reporting beneficial effects of psilocybin, known as expectancy bias. Thus, while the study by Moreno and colleagues (Moreno et al., 2006) demonstrates that psilocybin can produce beneficial effects on OCD symptoms in some patients, much more robust and well-controlled trials are needed to fully assess its efficacy (see “Discussion”).

### Surveys

Psychedelic use is illegal in most countries, limiting its availability to clinicians and researchers. As such, surveys and retrospective analyses of self-medication with psychedelics present interesting opportunities to understand how these drugs may benefit individuals with obsessive and/or compulsive symptoms. For example, a retrospective online survey found that patients with OCD self-medicating with psilocybin and LSD reported their treatment to be effective, and that the dose of the drug being taken correlated with the intensity of the acute effects and a reduction in symptom severity (Buot et al., 2023). Another survey found that the subjective effects of psychedelics, for example, the mystical experiences and psychological insight they produce, predicted self-reported reductions in obsessions and compulsions (Moreton et al., 2023). While it is difficult to ascertain the exact drugs and doses used in such reports, and retrospective surveys inevitably evoke recall bias, they support the clinical data that psychedelics may be useful for the treatment of OCD.

## THEORIES OF PSYCHEDELIC MECHANISM OF ACTION

### Modulation of the 5-HT System

OCD has historically been seen as a disease of the 5-HT system. Genetic studies have identified polymorphisms in the 5-HT transporter (5-HTT) and 5-HT_2A_ receptor in patients with OCD (Taylor et al., 2016), and alterations in peripheral markers of 5-HT function have also been reported (Delgado and Moreno, 1998a, 1998b). Moreover, SSRIs are successful treatments for some OCD patients (Jenike, 1993; El Mansari and Blier, 2006), implying that disruption of the 5-HT system may contribute to the pathophysiology of OCD.

Psilocybin and other classical, also known as serotonergic, psychedelics act on the 5-HT system, and in some regards resemble the effects of SSRIs (Goodman et al., 1989a; Pittenger, 2015). Classical psychedelics agonize 5-HT_2A/2C_ receptors, which underlie the hallucinogenic effects of psychedelics (Moreno and Delgado, 1997; Madsen et al., 2019; Andersen et al., 2021). Although data are lacking on how psychedelics alter 5-HT levels or transmission in people with OCD, it is hypothesized that they improve OCD symptoms by potentiating 5-HT_2A_-mediated signaling to alleviate existing deficits. Moreover, anecdotal evidence suggests that, when taken alone, 5-HT_2A_ antagonists such as clozapine and metergoline can exacerbate OCD symptoms (Moreno and Delgado, 1997; El Mansari and Blier, 2006). Together, these studies suggest that the 5-HT_2A_ receptor may be involved in the emergence of OCD symptoms, and that psychedelics act, at least acutely, by agonizing these receptors.

Preclinical studies also suggest that altered 5-HT signaling may ameliorate OCD symptoms (for a review of animal models of OCD, see Pittenger et al., 2017). One of the most widely used rodent models of OCD is marble burying and digging behavior. In this model, glass marbles are placed in a cage lined with sawdust bedding, and mice will dig to cover over the marbles. While this digging is a naturalist exploratory behavior (Deacon, 2006; Pond et al., 2021), it can also be thought of as a compulsive behavior (Angoa-Pérez et al., 2013). As such, typical pharmacological treatments for OCD reduce digging in the marble burying test (Thomas et al., 2009; Singh et al., 2023). Psychedelics produce similar effects—for example, acute psilocybin treatment reduced marble burying in mice compared to saline-treated controls (Matsushima et al., 2009; Singh et al., 2023). These effects likely occur via 5-HT_2A/2C_ receptor modulation, as experimental 5-HT_2A_ agonists such as 2,5-dimethoxy-4-iodoamphetamine (DOI) and 4-(2-((2-hydroxybenzyl)amino)ethyl)-2,5-dimethoxybenzonitrile reduced digging in mice (Egashira et al., 2012; Jensen et al., 2020; Odland et al., 2021b). Moreover, mice lacking 5-HT_2C_ receptor expression show compulsive digging in the marble burying test (Chou-Green et al., 2003). Preclinical studies therefore agree with clinical observations that classical psychedelics may reduce compulsive behaviors via altered 5-HT_2A/2C_ receptor signaling.

Yet, understanding the mechanism of action of classical psychedelics is complicated by their nonselective pharmacology, with several studies pointing away from the 5-HT_2A/2C_ receptors as the mediators of symptomatic improvement. For example, coadministration of the 5-HT_2A_ antagonist M100907 did not block the reduction in digging produced by either psilocybin or DOI in mice (Odland et al., 2021a; Singh et al., 2023), suggesting other receptors could be involved. For instance, the effects of psilocybin on marble burying in mice were augmented by 8-OH-DPAT, a 5-HT_1A_ receptor agonist (Singh et al., 2023). Moreover, LSD had no effect on grooming behaviors, another potential correlate of compulsive behaviors, in heterozygous 5-HTT depletion mice, implicating a role of 5-HT reuptake in its mechanism of action (Kyzar et al., 2016). Clinical data also suggest that other 5-HT_2C_ receptor agonists may exacerbate OCD symptoms—in 2 case reports, MDMA, a 5-HT releasing agent and 5-HT_2C_ agonist, induced OCD symptoms in long-time users (Marchesi et al., 2009). Thus, while 5-HT_2A/2C_ receptors appear to be the primary targets of psychedelics, there is no clear consensus on the relationship between 5-HT receptor pharmacology and reduced OCD symptomatology.

### Normalizing Glutamatergic Dysregulation and Cortico-Striatal-Thalamo-Cortical Hyperactivity

Glutamate dysregulation may contribute to the pathology of OCD (Pittenger et al., 2011; Pittenger, 2015), and is seen across the obsessive–compulsive spectrum (Martinotti et al., 2021). Glutamate activates the cortico-striatal-thalamo-cortical (CSTC) circuit, in which activity is elevated in patients with OCD (Menzies et al., 2008; Tang et al., 2016; Nasir et al., 2020). Hyperactivity in this circuit has been hypothesized to cause irrational fears or obsessions, as well as habitual actions that appear inappropriate for the environment, such as repetitive or compensatory behaviors (Calzà et al., 2019; Ehrmann et al., 2022). The extent of CSTC hyperactivity correlates with OCD severity (Zambrano-Vazquez and Allen, 2014).

Psychedelics also modulate glutamatergic transmission (Aghajanian and Marek, 1999; Fineberg and Robbins, 2021), with mice lacking the metabotropic glutamate receptor 2 rendered insensitive to cellular or behavioral responses to LSD (Moreno et al., 2011). Moreover, all successful therapies for OCD show normalization of CSTC circuits (Saxena et al., 1996; Van der Straten et al., 2017). 5-HT_2A_ agonists, including LSD, acutely disrupt activity in the CSTC circuit by reducing the influence of the striatum on the thalamus and increase thalamocortical connectivity with sensorimotor cortices (Müller et al., 2017; Preller et al., 2018; Avram et al., 2021; Delli Pizzi et al., 2023). This lessens sensory input filtering of the thalamus, which is thought to be excessive in OCD (Nichols, 2004; De Gregorio et al., 2018; Preller et al., 2019). The long-term effects of psychedelics on CSTC connectivity, and the effects of psychedelics on CSTC circuits in patients with OCD, have yet to be studied. Nonetheless, normalization of activity these networks is hypothesized to contribute to their beneficial clinical effects.

### Normalizing Orbitofrontal Cortex Activity

Positron emission tomography studies have implicated hyperactivity in the orbitofrontal cortex (OFC) in the pathophysiology of OCD (Baxter et al., 1987; Saxena et al., 1996; El Mansari and Blier, 2006). Classical pharmacotherapy and behavioral therapies for OCD normalize this elevated activity (Benkelfat et al., 1990; Brody et al., 1998; Bergqvist et al., 1999b)—for instance, patients that respond to SSRIs see a reduction in metabolic activity in OFC (Bergqvist et al., 1999a). Psilocybin may work in a similar manner, as an acute dose reduced blood-oxygen-level-dependent signal in the fronto-temporal-parietal cortical regions in healthy volunteers (Carhart-Harris et al., 2012b). This effect has been corroborated in electrophysiological studies in rats, where DOI suppressed activity in the OFC (Bergqvist et al., 1999b). Microiontophoretic injections of LSD into the OFC also attenuated the firing of OFC neurons, as well as enhancing the effects of co-applied 5-HT due to agonism of 5-HT_2C_ receptors (Zghoul and Blier, 2003). These studies therefore indicate that the OFC may be a key target brain region for psychedelics in the amelioration of OCD symptoms, at least when given acutely. There have yet to be studies on any persistent or long-term changes in OFC connectivity following single or repeated doses of any psychedelic.

### Enhanced Neuroplasticity

The half-lives of most psychedelics are relatively short (2.6-4.0 hours LSD, 2-3 hours psilocybin) (Brown et al., 2017; Dolder et al., 2017; Holze et al., 2023, 2024; Ley et al., 2023); yet their behavioral effects may last for several days and even weeks. It is therefore likely that psychedelics induce neuroadaptive changes in the brain to mediate these long-term effects. Evidence of enhanced plasticity mostly comes from rodent studies, where a single dose of LSD increased the expression of genes involved in synaptic plasticity, glutamatergic signaling, and cytoskeletal architecture (Nichols and Sanders-Bush, 2002). LSD was shown to induce synaptogenesis and the number of dendritic spines in primary rat cortical neurons and in the mouse prefrontal cortex in vivo (Ly et al., 2018; Shao et al., 2021). Increased hippocampal neurogenesis, increased density of dendritic spines in the frontal cortex, and elevated gene expression for synaptic assembly proteins were also observed 7 days after DOI exposure in mice (Catlow et al., 2013). Clinical data to support these findings in patients are sparse; yet, psilocybin therapy for patients with depression increased cognitive flexibility for 4 weeks following its administration (Doss et al., 2021), suggesting enhanced neuroplasticity. Psilocybin was also shown to acutely increase psychological flexibility, which mediated improvements in anxiety and depression (Davis et al., 2020). Increased brain-wide resting state connectivity, potentially in emotionally relevant brain areas, has also been reported for up to a month after acute psilocybin administration (Barrett et al., 2020). Moreover, microdosing with 10 µg LSD for 6 weeks produced some evidence of increased cortical plasticity in healthy male subjects compared to placebo-treated controls (Murphy et al., 2024), although further studies are needed to determine if LSD would have such an effect in OCD patients or at other doses.

Psychedelics may also accelerate fear extinction (Vaidya et al., 1997). For example, LSD reversed deficits in avoidance learning in rats and primates (Roberts and Bradley, 1967; King et al., 1974), and psilocybin and LSD accelerated acute extinction of conditioned fear responses compared to saline-treated control mice (Catlow et al., 2013). These changes may reflect a more flexible brain after psilocybin exposure, which could interact with psychological therapy to enhance clinical responses (Ehrmann et al., 2022).

### Modulation of the Default Mode Network

The default mode network (DMN) is a group of brain regions that show high degrees of connectivity at rest and is activated by with spontaneous self-generated thought, internally directed cognition, and meta-cognition (Buckner et al., 2008; Goodkind et al., 2015; Soto et al., 2018; Modlin et al., 2023). The DMN controls how information is processed in lower-order brain regions, and gates information transmitted to the cortex. OCD is associated with changes in DMN activity, in that rigid and overlearned behavioral patterns arise in response to incongruent information due to issues with internally generated perception (Beucke et al., 2014; Gonçalves et al., 2017; Koch et al., 2018).

Functional magnetic resonance imaging (fMRI) studies show that psychedelics modulate the DMN (Carhart-Harris et al., 2012b, 2017). Psychedelics cause the temporary disintegration of resting state networks and are thought to “reset” their maladaptive activity (Carhart-Harris et al., 2016b; Carhart-Harris and Nutt, 2017; Carhart-Harris, 2019). This includes, for example, a reduction in blood flow within the DMN, and particularly the cingulate cortex, after psilocybin treatment (Carhart-Harris et al., 2012a, 2017; Barrett et al., 2020). This may allow for healthier engagement with the environment and ultimately the alteration of compulsive responses (Carhart-Harris, 2019; Madsen et al., 2020). Persistent changes in resting state connectivity have been reported for up to a month after psilocybin treatment (Barrett et al., 2020; McCulloch et al., 2022). Thus, the normalization of DMN activity may be responsible for the clinical benefits experienced by patients with mental health disorders, although this has not yet been demonstrated specifically in OCD patients.

The effects of psychedelics on the DMN may also involve altered 5-HT signaling and enhanced serotonergic connectivity with the DMN (Grandjean et al., 2021). One contemporary explanation that integrates the pharmacological and cognitive theories of psychedelics on OCD symptoms is the Relaxed Beliefs under Psychedelics and the Anarchic Brain (REBUS) theory. According to this model, psychedelics act via 5-HT_2A_ receptors in the cortex, such as in the OFC, to relax top-down constraints on emotions, cognition, and sensory perceptions, and to reduce DMN activity. This decreases the influence of prior beliefs and relieves the inhibition of bottom-up information signaling from subcortical regions, for example, the thalamus (Carhart-Harris and Friston, 2019; Singleton et al., 2022). Further evidence for this theory is discussed in Doss et al. (2022). Thus, in OCD when there is excessive cortical influence on information gating, psychedelics could reduce this input, normalizing information flow in the CSTC (Vollenweider et al., 1998; Preller et al., 2018).

### Long-Term Psychological Effects

The subjective experiences produced by psychedelics, such as powerful insights or profound spiritual and existential realizations, may also play a role in treatment responses (Moreno et al., 2006). One study of patients with depression found that the acute mystical experiences produced by psilocybin correlated with symptomatic improvement (Carhart-Harris and Nutt, 2017). Others have also found a link between the acute effects of psilocybin and symptomatic improvements for depression and anxiety (Griffiths et al., 2011, 2016; Roseman et al., 2018; Davis et al., 2020). It has been postulated that the thoughts experienced during psychedelic exposure may provide new insight into an individual’s illness and lead them to discover new ways of responding to their inner experiences (Kelmendi et al., 2022; Tiwari et al., 2024). Thus, the insights produced during these experiences may synergize with the molecular and network-level changes to produce real changes in how patients perceive and respond to the world and consequently their symptoms.

Yet, it is also feasible that symptomatic improvements are artifacts of mindset and setting, in that the context or pleasurable experience of psychedelics themselves may simply distract from OCD symptoms (Moreno et al., 2006). Critiques of the use of psychoactive compounds for mental health disorders have suggested that the environment (“setting”) of their administration, for example, the clinical setting and calming atmosphere, and the mindset (“set”) of the patient going into the experience, may relate to their apparent symptomatic improvements, rather than their direct pharmacological effects (Martinotti et al., 2021). On the other hand, some patients still see clinical improvements to their OCD symptoms despite not experiencing hallucinations (Wilcox, 2014; Lugo-Radillo and Cortes-Lopez, 2021), or after developing tolerance to the acute psychedelic effects (Moreno and Delgado, 1997). Some participants even found it an actively stressful or unpleasant experience (Moreno et al., 2006), yet still report improvements to symptoms. The beneficial effects of psychedelics for OCD are therefore not solely attributable to their acute or pleasant psychological effects.

## DISCUSSION

Evidence is accumulating for the efficacy of psychedelics, and especially psilocybin, for OCD. We are also rapidly advancing our knowledge of their mechanisms of action, with hypotheses including changes to 5-HT transmission, altered CSTC and OFC activity, and enhanced neuroplasticity. Yet, many questions remain about the use of psychedelic therapy for OCD.

### Evaluating the Efficacy of Psychedelics for OCD

Published studies suggest that psychedelics may have some efficacy in treating obsessive and/or compulsive symptoms in patients with OCD. Nonetheless, the published studies do not systemically assess the effects of psychedelics on OCD symptoms, and as such, the optimal dose, duration, and frequency of psychedelic administration have yet to be established (Kelmendi et al., 2022). The lack of long-term follow-up in the current studies also limits our understanding of the persistent effects of psychedelics on OCD. For instance, Moreno and colleagues only measured Y-BOCS scores for 24 hours after each dose, despite patients reporting persistent improvements up to a week later (Moreno et al., 2006). Ongoing clinical trials, such as trial NCT03300947 in the United States, will collect Y-BOCS score for 6 months after blinded and repeated psilocybin administration to assess its long-term effects more rigorously (Ehrmann et al., 2022). Another will look at the effects of repeated psilocybin dosing in OCD (NCT05370911). Similarly, a trial established at Yale University is currently recruiting participants with treatment-resistant OCD (NCT03356483) and will involve a 12-week follow-up period to understand the duration of effects of acute double-blind, placebo-controlled trial for the oral administration of psilocybin (Kelmendi et al., 2022; Ching et al., 2023).

It is also not clear what the target population for psychedelic therapy should be; certain subgroups of OCD patients may be more suited to this treatment approach than others. So far, participation has been based on whether patients have previously experienced positive effects of psychedelics on their symptoms. This makes it more likely that they will tolerate the experience in a clinical setting (Moreno et al., 2006; Kelmendi et al., 2022), but may have biased the existing data toward positive responses. It would therefore be useful to identify biomarkers that predict the likelihood of a patient responding to psychedelic treatment. For example, psilocybin reduced associative and brain-wide connectivity in fMRI scans of healthy volunteers, and data from these scans could identify the individuals that displayed the strongest psychological responses to psilocybin (Preller et al., 2020). If applied to patients, preemptive brain scans could be used to identify those most likely to respond to psychedelic therapy. This may be particularly relevant for patients with treatment-resistant OCD who have the most to gain from novel therapy options. Moreover, given the clinical use of psychedelics is likely to be incredibly expensive, it will likely prove necessary to be selective in the patients that undertake this treatment.

There may also be other factors that influence individual responses to psychedelic therapy that are not typically considered during participant selection. For instance, an individual’s preparation, expectations of the experience, and attitude toward psychedelics may interact with their acute effects to alter their subjective experience, including its intensity and pleasantness, and thus the overall efficacy (Hartogsohn, 2017; Ehrmann et al., 2022; Modlin et al., 2023). It may also be important to consider an individual’s interactions with the therapist or guide present during treatment administration, as well as in any subsequent integration sessions (Carhart-Harris et al., 2018; Nutt, 2019; Andersen et al., 2021) in the treatment response. Thus, we need to develop quantitative measures to assess a person’s readiness, motivation, and capacity for psychedelic treatment (Modlin et al., 2023), to more accurately assess their efficacy.

It is also difficult to assess the efficacy of psychedelics from the current data due to the small scale of the trials (for instance, just 9 participants in Moreno et al., 2006), thus making them underpowered. Such trials are also limited by their narrow inclusion criteria, despite OCD being a highly heterogeneous (see “Introduction”) and with extensive comorbidities. Ongoing trials aim to increase the number of OCD patients undergoing psilocybin therapy to further assess its efficacy (eg, NCT05546658). Nevertheless, it may be more useful to probe the effects of psychedelics across mental health disorders using transdiagnostic markers such as anxiety and fear, rather than focusing on disease-specific symptoms (Kelly et al., 2021). Identifying unitary biomarkers of mental health that transcend diagnoses will allow us to retrospectively analyze previous experiments to identify potential beneficial effects of psychedelics for OCD, but also to be able to include more participants in future trials.

Finally, comparative studies are needed to assess the efficacy of psychedelics relative to current treatments for OCD. This will be particularly important for patients with treatment-resistant OCD, for whom the current treatments are not working. The existing evidence is also primarily for the effects of psilocybin for OCD, since this is the substance most studied in the modern era. Therefore, other psychedelic compounds may be just as promising but not studied; future research should broaden the range of psychoactive compounds included in clinical research to identify those that have the greatest potential benefits for OCD.

### Limited Understanding of the Mechanism of Action

Generally, evidence for the mechanism of action of psychedelics is circumstantial, even for disorders that have been more heavily studied, such as anxiety and depression (see review by Tiwari et al., 2024). Studies of OCD typically suggest that psychedelics must reverse some underlying disruption in the brain, although they heavily relying on data from healthy volunteers or those with other mental health conditions. Consequently, we lack specific data of the effects of psychedelics on brain neurochemistry and connectivity in patients with OCD. An ongoing trials (NCT03300947) will address this knowledge gap by performing fMRI on patients before and after psilocybin administration and compared to lorazepam treatment (Ehrmann et al., 2022; Kelmendi et al., 2022; Ching et al., 2023). It is hoped that these data will shed light on changes to functional connectivity in the brain during psilocybin administration, particularly how the DMN and other resting state networks may be affected. Another trial (NCT06258031) will combine electroencephalography and cognitive tasks to better understand changes in cognitive flexibility and neuroplasticity following 2 exposures to psilocybin in patients with OCD.

The current animal models of OCD also have poor predictive validity, making it hard to draw conclusions about the molecular mechanisms of psychedelics for compulsive behaviors. For example, anxiolytic drugs with no anti-compulsive effects in humans can reduce marble burying in rodents (Taylor, 2017; Thompson and Dulawa, 2017). Alternative models of compulsive behaviors involve rats learning to habitually press a lever in response to a signal to administer a food reward; even after the signal is attenuated, animals will continue to compulsively press the lever (Flaisher-Grinberg et al., 2008). However, this model does not reflect the anti-compulsive effects of DOI (Flaisher-Grinberg et al., 2008). We still lack models for the more subjective symptoms of OCD, for example, intrusive thoughts, which ultimately may not be possible to study in animals (Pittenger et al., 2017). To advance our understanding of the mechanisms of action of psychedelics, we need to develop novel animal models with better face, construct, and predictive validity for OCD.

Finally, we do not understand how psychological therapy may interact with psychedelic experiences to mediate improvements in OCD symptoms. As the existing studies did not follow standardized protocols, some of the variation in their results may be attributable to the type of psychological support or therapy provided. Future trials of psilocybin and other psychedelics should therefore compare structured psychological support as part of the treatment protocol to psychedelic administration alone to determine the roles of each aspect in the therapeutic response. Notably, many of the ongoing and proposed trials for psilocybin for OCD will include some form of therapy alongside drug administration—for instance, trial NCT03300947 will integrate 8 therapy sessions into the dosing regimen to help patients understand the insights they gain during the acute psychedelic effects, and another (NCT04882839) will involve 15 therapeutic sessions, 12 therapy sessions, and three 8-hour experimental sessions under the influence of psilocybin (Ching et al., 2023).

In general, there are many ongoing questions about the mechanisms by which psychedelics act to improve OCD symptoms. Nonetheless, it is unlikely that psychedelics act via a single mechanism to have their therapeutic effects (Tiwari et al., 2024), necessitating further clinical and preclinical studies with a variety of technical and theoretical approaches. A notable limitation of this review, however, is that it is not systematic; as such, some studies will not have been discussed, particularly regarding preclinical studies of psychedelics and the broader literature on the mechanisms of psychedelics for the treatment of anxiety and depression.

### Issues With Using Psychedelics to Treat OCD

There are several issues with using psychedelics as standard treatments for OCD that must also be considered. Firstly, it is incredibly difficult to blind and control studies of psychedelics due to their robust psychoactive and hallucinogenic properties, even at very low doses (Butler et al., 2022). The open-label nature of many of these trials also leaves participants vulnerable to expectancy bias (Tiwari et al., 2024). Trials and surveys of psychedelic use also tend to attract people who are open to the possibility that these drugs will work. For example, a 2023 survey of psychedelic use for OCD recruited responders by publicizing the survey on OCD forums and online pages for psychedelic societies and users (Buot et al., 2023). This will have biased patient samples toward those who are more open to the idea of them being useful treatments, further exaggerating augmented effect sizes produced from poor blinding. To tackle these issues, an ongoing trial of psilocybin (NCT03300947) will use the anxiolytic lorazepam as an active placebo to make it more difficult for patients to identify whether they have received psilocybin or control in a treatment session (Ehrmann et al., 2022). This trial will also include psychedelic-naïve patients to confirm the generalizability of the earlier findings. Other studies will also include double-blinded stages (NCT03356483) to overcome some of the limitations of previous studies.

Particular care is needed to identify and exclude patients from treatment for whom psychedelics may cause further harm. In a trial for ketamine for OCD, for instance, ketamine worsened dysphoria, anxiety, and suicidality in 3 patients with psychological comorbidities (Bloch et al., 2012; Niciu et al., 2013). Psychedelics are also thought to exacerbate preexisting psychoses (Johnson et al., 2008), so this treatment approach may not be suitable for patients with a history of schizophrenia. Given that as many as 90% of patients with OCD have comorbidities (Ruscio et al., 2010), we need a better understanding of how to dissociate the beneficial effects of psychedelics from those that may cause more long-term harm or perpetuate the symptoms.

Finally, the legal restrictions on psychedelics in many countries make it difficult to assess the true scale of their use and benefits for psychiatric disorders, as well as making scientific studies of their utility highly regulated and costly. This is despite evidence that there is no association between recreational psychedelic use and mental health issues; in fact, suicidality and mental health problems were lower in psychedelic users than in nonusers (Grinspoon and Bakalar, 1981; Johansen and Krebs, 2015). They also pose a low risk of dependency than other psychoactive compounds (Rucker et al., 2016; Nutt, 2019; Castro Santos and Gama Marques, 2021). Researchers and clinicians alike are therefore calling for regulatory change to facilitate the further exploration of the clinical potential of psychedelic-assisted therapy (Nutt, 2019; Kelmendi et al., 2022; Bhuiya et al., 2023).

## CONCLUSION

Psychedelics, and particularly psilocybin, are emerging as novel and potentially useful treatments for OCD, and early data suggest that they are safe and well-tolerated. Compared to the current therapeutic approaches, psychedelics offer faster treatment responses with no need for daily administration, which would increase compliance. Nonetheless, the current data are limited to case reports and small clinical trials with methodological issues including poor control groups and lack of follow-up, meaning the long-term effects of psychedelic therapy remain unclear. Further studies should aim to conclusively demonstrate the efficacy of psychedelics for OCD by focusing on transdiagnostic biomarkers to link with studies with other mental health conditions. The integration of psychedelic experiences with psychotherapy may increase the efficacy of this therapeutic approach, and thus should become commonplace in further studies of OCD. Future trials will also include the use of brain imaging techniques to better understand the biological effects of psychedelics. This will advance our knowledge of the mechanisms of psychedelics and how they may be useful for the treatment of OCD, as well as lead to the development of novel therapeutic approaches.

## Data Availability

No new data were generated or analyzed in support of this research.

## References

[CIT0001] Aghajanian GK , Marek GJ (1999) Serotonin and hallucinogens. Neuropsychopharmacology 21:16S–23S. 10432484 10.1016/S0893-133X(98)00135-3

[CIT0002] Andersen KAA , Carhart-Harris R, Nutt DJ, Erritzoe D (2021) Therapeutic effects of classic serotonergic psychedelics: a systematic review of modern-era clinical studies. Acta Psychiatr Scand 143:101–118. 33125716 10.1111/acps.13249

[CIT0003] Angoa-Pérez M , Kane MJ, Briggs DI, Francescutti DM, Kuhn DM (2013) Marble burying and nestlet shredding as tests of repetitive, compulsive-like behaviors in mice. J Vis Exp 82:50978. 10.3791/50978 PMC4108161 24429507

[CIT0004] Avram M , Rogg H, Korda A, Andreou C, Müller F, Borgwardt S (2021) Bridging the gap? Altered thalamocortical connectivity in psychotic and psychedelic states. Front Psychiatry 12:706017. 34721097 10.3389/fpsyt.2021.706017 PMC8548726

[CIT0005] Bakalar JB , Grinspoon L (1990) Testing psychotherapies and drug therapies: the case of psychedelic drugs. In: Ecstasy: the clinical, pharmacological and neurotoxicological effects of the drug MDMA (Peroutka SJ, ed), pp 37–52. Boston, MA: Springer US.

[CIT0006] Bandeira ID , Lins-Silva DH, Cavenaghi VB, Dorea-Bandeira I, Faria-Guimarães D, Barouh JL, Jesus-Nunes AP, Beanes G, Souza LS, Leal GC, Sanacora G, Miguel EC, Sampaio AS, Quarantini LC (2022) Ketamine in the treatment of obsessive-compulsive disorder: a systematic review. Harv Rev Psychiatry 30:135–145. 35267254 10.1097/HRP.0000000000000330

[CIT0007] Barrett FS , Doss MK, Sepeda ND, Pekar JJ, Griffiths RR (2020) Emotions and brain function are altered up to one month after a single high dose of psilocybin. Sci Rep 10:2214. 32042038 10.1038/s41598-020-59282-y PMC7010702

[CIT0008] Baxter LR Jr , Phelps ME, Mazziotta JC, Guze BH, Schwartz JM, Selin CE (1987) Local cerebral glucose metabolic rates in obsessive-compulsive disorder. A comparison with rates in unipolar depression and in normal controls. Arch Gen Psychiatry 44:211–218. 3493749 10.1001/archpsyc.1987.01800150017003

[CIT0009] Belayachi S , Van der Linden M (2017) The cognitive heterogeneity of obsessive-compulsive checking. J Cogn Educ Psychol 16:9–22.

[CIT0010] Benkelfat C , Nordahl TE, Semple WE, King AC, Murphy DL, Cohen RM (1990) Local cerebral glucose metabolic rates in obsessive-compulsive disorder. Patients treated with clomipramine. Arch Gen Psychiatry 47:840–848. 2393342 10.1001/archpsyc.1990.01810210048007

[CIT0011] Bergqvist PB , Bouchard C, Blier P (1999a) Effect of long-term administration of antidepressant treatments on serotonin release in brain regions involved in obsessive-compulsive disorder. Biol Psychiatry 45:164–174. 9951563 10.1016/s0006-3223(98)00154-1

[CIT0012] Bergqvist PBF , Dong J, Blier P (1999b) Effect of atypical antipsychotic drugs on 5-HT2 receptors in the rat orbito-frontal cortex: an in vivo electrophysiological study. Psychopharmacology (Berl) 143:89–96. 10227084 10.1007/s002130050923

[CIT0013] Beucke JC , Sepulcre J, Eldaief MC, Sebold M, Kathmann N, Kaufmann C (2014) Default mode network subsystem alterations in obsessive-compulsive disorder. Br J Psychiatry 205:376–382. 25257066 10.1192/bjp.bp.113.137380

[CIT0014] Bhuiya NMA , Jacobs RJ, Wang K, Sun Y, Nava B, Sampiere L, Yerubandi A, Caballero J (2023) Predictors of pharmacy students’ attitudes about the therapeutic use of psilocybin. Cureus 15:e45169. 37842360 10.7759/cureus.45169 PMC10575550

[CIT0015] Bloch MH , Wasylink S, Landeros-Weisenberger A, Panza KE, Billingslea E, Leckman JF, Krystal JH, Bhagwagar Z, Sanacora G, Pittenger C (2012) Effects of ketamine in treatment-refractory obsessive-compulsive disorder. Biol Psychiatry 72:964–970. 22784486 10.1016/j.biopsych.2012.05.028 PMC3667652

[CIT0016] Bragdon LB , Coles ME (2017) Examining heterogeneity of obsessive-compulsive disorder: evidence for subgroups based on motivations. J Anxiety Disord 45:64–71. 27960103 10.1016/j.janxdis.2016.12.002

[CIT0017] Brandrup E , Vanggaard T (1977) LSD treatment in a severe case of compulsive neurosis. Acta Psychiatr Scand 55:127–141. 842386 10.1111/j.1600-0447.1977.tb00149.x

[CIT0018] Brody AL , Saxena S, Schwartz JM, Stoessel PW, Maidment K, Phelps ME, Baxter LR Jr (1998) FDG-PET predictors of response to behavioral therapy and pharmacotherapy in obsessive–compulsive disorder. Psychiatry Res 84:1–6. 9870412 10.1016/s0925-4927(98)00041-9

[CIT0019] Brown RT , Nicholas CR, Cozzi NV, Gassman MC, Cooper KM, Muller D, Thomas CD, Hetzel SJ, Henriquez KM, Ribaudo AS, Hutson PR (2017) Pharmacokinetics of escalating doses of oral psilocybin in healthy adults. Clin Pharmacokinet 56:1543–1554. 28353056 10.1007/s40262-017-0540-6

[CIT0020] Buckner RL , Andrews-Hanna JR, Schacter DL (2008) The brain’s default network: anatomy, function, and relevance to disease. Ann N Y Acad Sci 1124:1–38. 18400922 10.1196/annals.1440.011

[CIT0021] Buot A , Pallares C, Oganesyan A, Dauré C, Bonnelle V, Burguière E, Dos Santos JFA, N’Diaye K, Ljuslin M, Smith P, Verroust V, Wyplosz B, Morgiève M, Mallet L (2023) Improvement in OCD symptoms associated with serotoninergic psychedelics: a retrospective online survey. Sci Rep 13:13378. 37591906 10.1038/s41598-023-39812-0 PMC10435518

[CIT0022] Butler M , Jelen L, Rucker J (2022) Expectancy in placebo-controlled trials of psychedelics: if so, so what? Psychopharmacology (Berl) 239:3047–3055. 36063208 10.1007/s00213-022-06221-6 PMC9481484

[CIT0023] Calzà J , Gürsel DA, Schmitz-Koep B, Bremer B, Reinholz L, Berberich G, Koch K (2019) Altered cortico–striatal functional connectivity during resting state in obsessive–compulsive disorder. Front Psychiatry 10:319. 31133898 10.3389/fpsyt.2019.00319 PMC6524661

[CIT0024] Carhart-Harris RL (2019) How do psychedelics work? Curr Opin Psychiatry 32:16–21. 30394903 10.1097/YCO.0000000000000467

[CIT0025] Carhart-Harris RL , Friston KJ (2019) REBUS and the anarchic brain: toward a unified model of the brain action of psychedelics. Pharmacol Rev 71:316–344. 31221820 10.1124/pr.118.017160 PMC6588209

[CIT0026] Carhart-Harris RL , Nutt D (2017) Serotonin and brain function: a tale of two receptors. J Psychopharmacol 31:1091–1120. 28858536 10.1177/0269881117725915 PMC5606297

[CIT0027] Carhart-Harris RL , Leech R, Williams TM, Erritzoe D, Abbasi N, Bargiotas T, Hobden P, Sharp DJ, Evans J, Feilding A, Wise RG, Nutt DJ (2012a) Implications for psychedelic-assisted psychotherapy: functional magnetic resonance imaging study with psilocybin. Br J Psychiatry 200:238–244. 22282432 10.1192/bjp.bp.111.103309

[CIT0028] Carhart-Harris RL , Erritzoe D, Williams T, Stone JM, Reed LJ, Colasanti A, Tyacke RJ, Leech R, Malizia AL, Murphy K, Hobden P, Evans J, Feilding A, Wise RG, Nutt DJ (2012b) Neural correlates of the psychedelic state as determined by fMRI studies with psilocybin. Proc Natl Acad Sci U S A 109:2138–2143. 22308440 10.1073/pnas.1119598109 PMC3277566

[CIT0029] Carhart-Harris RL , Bolstridge M, Rucker J, Day CMJ, Erritzoe D, Kaelen M, Bloomfield M, Rickard JA, Forbes B, Feilding A, Taylor D, Pilling S, Curran VH, Nutt DJ (2016a) Psilocybin with psychological support for treatment-resistant depression: an open-label feasibility study. Lancet Psychiatry 3:619–627. 27210031 10.1016/S2215-0366(16)30065-7

[CIT0030] Carhart-Harris RL , et al (2016b) Neural correlates of the LSD experience revealed by multimodal neuroimaging. Proc Natl Acad Sci U S A 113:4853–4858. 27071089 10.1073/pnas.1518377113 PMC4855588

[CIT0031] Carhart-Harris RL , Roseman L, Bolstridge M, Demetriou L, Pannekoek JN, Wall MB, Tanner M, Kaelen M, McGonigle J, Murphy K, Leech R, Curran HV, Nutt DJ (2017) Psilocybin for treatment-resistant depression: fMRI-measured brain mechanisms. Sci Rep 7:13187. 29030624 10.1038/s41598-017-13282-7 PMC5640601

[CIT0032] Carhart-Harris RL , Bolstridge M, Day CMJ, Rucker J, Watts R, Erritzoe DE, Kaelen M, Giribaldi B, Bloomfield M, Pilling S, Rickard JA, Forbes B, Feilding A, Taylor D, Curran HV, Nutt DJ (2018) Psilocybin with psychological support for treatment-resistant depression: six-month follow-up. Psychopharmacology (Berl) 235:399–408. 29119217 10.1007/s00213-017-4771-x PMC5813086

[CIT0033] Carhart-Harris R , Giribaldi B, Watts R, Baker-Jones M, Murphy-Beiner A, Murphy R, Martell J, Blemings A, Erritzoe D, Nutt DJ (2021) Trial of psilocybin versus escitalopram for depression. N Engl J Med 384:1402–1411. 33852780 10.1056/NEJMoa2032994

[CIT0034] Castro Santos H , Gama Marques J (2021) What is the clinical evidence on psilocybin for the treatment of psychiatric disorders? A systematic review. Porto Biomed J 6:e128. 33884324 10.1097/j.pbj.0000000000000128 PMC8055489

[CIT0035] Catlow B , Song S, Paredes D, Kirstein C, Sanchez-Ramos J (2013) Effects of psilocybin on hippocampal neurogenesis and extinction of trace fear conditioning. Exp Brain Res 228:481–491. 23727882 10.1007/s00221-013-3579-0

[CIT0036] Ching THW , Grazioplene R, Bohner C, Kichuk SA, DePalmer G, D’Amico E, Eilbott J, Jankovsky A, Burke M, Hokanson J, Martins B, Witherow C, Patel P, Amoroso L, Schaer H, Pittenger C, Kelmendi B (2023) Safety, tolerability, and clinical and neural effects of single-dose psilocybin in obsessive-compulsive disorder: protocol for a randomized, double-blind, placebo-controlled, non-crossover trial. Front Psychiatry 14:1178529. 37181888 10.3389/fpsyt.2023.1178529 PMC10166878

[CIT0037] Chou-Green JM , Holscher TD, Dallman MF, Akana SF (2003) Compulsive behavior in the 5-HT2C receptor knockout mouse. Physiol Behav 78:641–649. 12782219 10.1016/s0031-9384(03)00047-7

[CIT0038] Davis AK , Barrett FS, Griffiths RR (2020) Psychological flexibility mediates the relations between acute psychedelic effects and subjective decreases in depression and anxiety. J Contextual Behav Sci 15:39–45. 32864325 10.1016/j.jcbs.2019.11.004 PMC7451132

[CIT0039] Davis AK , Barrett FS, May DG, Cosimano MP, Sepeda ND, Johnson MW, Finan PH, Griffiths RR (2021) Effects of psilocybin-assisted therapy on major depressive disorder: a randomized clinical trial. JAMA Psychiatry 78:481–489. 33146667 10.1001/jamapsychiatry.2020.3285 PMC7643046

[CIT0040] Deacon RM (2006) Digging and marble burying in mice: simple methods for in vivo identification of biological impacts. Nat Protoc 1:122–124. 17406223 10.1038/nprot.2006.20

[CIT0041] De Gregorio D , Enns JP, Nuñez NA, Posa L, Gobbi G (2018) d-Lysergic acid diethylamide, psilocybin, and other classic hallucinogens: mechanism of action and potential therapeutic applications in mood disorders. Prog Brain Res 242:69–96. 30471683 10.1016/bs.pbr.2018.07.008

[CIT0042] Delgado PL , Moreno FA (1998a) Different roles for serotonin in anti-obsessional drug action and the pathophysiology of obsessive-compulsive disorder. Br J Psychiatry 173:21–25. 9829023

[CIT0043] Delgado PL , Moreno FA (1998b) Hallucinogens, serotonin and obsessive-compulsive disorder. J Psychoactive Drugs 30:359–366. 9924841 10.1080/02791072.1998.10399711

[CIT0044] Delli Pizzi S , Chiacchiaretta P, Sestieri C, Ferretti A, Tullo MG, Della Penna S, Martinotti G, Onofrj M, Roseman L, Timmermann C, Nutt DJ, Carhart-Harris RL, Sensi SL (2023) LSD-induced changes in the functional connectivity of distinct thalamic nuclei. Neuroimage 283:120414. 37858906 10.1016/j.neuroimage.2023.120414

[CIT0045] Denys D (2006) Pharmacotherapy of obsessive-compulsive disorder and obsessive-compulsive spectrum disorders. Psychiatr Clin North Am 29:553–584, xi. 16650723 10.1016/j.psc.2006.02.013

[CIT0046] Denys D , van der Wee N, van Megen HJ, Westenberg HG (2003) A double-blind comparison of venlafaxine and paroxetine in obsessive-compulsive disorder. J Clin Psychopharmacol 23:568–575. 14624187 10.1097/01.jcp.0000095342.32154.54

[CIT0047] Dolder PC , Schmid Y, Steuer AE, Kraemer T, Rentsch KM, Hammann F, Liechti ME (2017) Pharmacokinetics and pharmacodynamics of lysergic acid diethylamide in healthy subjects. Clin Pharmacokinet 56:1219–1230. 28197931 10.1007/s40262-017-0513-9 PMC5591798

[CIT0048] Doss MK , Považan M, Rosenberg MD, Sepeda ND, Davis AK, Finan PH, Smith GS, Pekar JJ, Barker PB, Griffiths RR, Barrett FS (2021) Psilocybin therapy increases cognitive and neural flexibility in patients with major depressive disorder. Transl Psychiatry 11:574. 34750350 10.1038/s41398-021-01706-y PMC8575795

[CIT0049] Doss MK , Madden MB, Gaddis A, Nebel MB, Griffiths RR, Mathur BN, Barrett FS (2022) Models of psychedelic drug action: modulation of cortical-subcortical circuits. Brain 145:441–456. 34897383 10.1093/brain/awab406 PMC9014750

[CIT0050] Egashira N , Okuno R, Shirakawa A, Nagao M, Mishima K, Iwasaki K, Oishi R, Fujiwara M (2012) Role of 5-hydroxytryptamine_2C_ receptors in marble-burying behavior in mice. Biol Pharm Bull 35:376–379. 22382324 10.1248/bpb.35.376

[CIT0051] Ehrmann K , Allen JJB, Moreno FA (2022) Psilocybin for the treatment of obsessive-compulsive disorders. Curr Top Behav Neurosci 56:247–259. 34784024 10.1007/7854_2021_279

[CIT0052] El Mansari M , Blier P (2006) Mechanisms of action of current and potential pharmacotherapies of obsessive-compulsive disorder. Prog Neuropsychopharmacol Biol Psychiatry 30:362–373. 16427729 10.1016/j.pnpbp.2005.11.005

[CIT0053] Evans JW , Szczepanik J, Brutsché N, Park LT, Nugent AC, Zarate CA Jr (2018) Default mode connectivity in major depressive disorder measured up to 10 days after ketamine administration. Biol Psychiatry 84:582–590. 29580569 10.1016/j.biopsych.2018.01.027 PMC6093808

[CIT0054] Fineberg N , Robbins TW (2021) The neurobiology and treatment of OCD: accelerating progress. Berlin/Heidelberg, Germany: Springer.

[CIT0055] Fineberg NA , Bullock T, Montgomery DB, Montgomery SA (1992) Serotonin reuptake inhibitors are the treatment of choice in obsessive–compulsive disorder. Int Clin Psychopharmacol 7:43–47. 1517558 10.1097/00004850-199206001-00012

[CIT0056] Flaisher-Grinberg S , Klavir O, Joel D (2008) The role of 5-HT2A and 5-HT2C receptors in the signal attenuation rat model of obsessive-compulsive disorder. Int J Neuropsychopharmacol 11:811–825. 18339223 10.1017/S146114570800847X

[CIT0057] Gonçalves OF , Soares JM, Carvalho S, Leite J, Ganho-Ávila A, Fernandes-Gonçalves A, Pocinho F, Carracedo A, Sampaio A (2017) Patterns of default mode network deactivation in obsessive–compulsive disorder. Sci Rep 7:44468. 28287615 10.1038/srep44468 PMC5347382

[CIT0058] Goodkind M , Eickhoff SB, Oathes DJ, Jiang Y, Chang A, Jones-Hagata LB, Ortega BN, Zaiko YV, Roach EL, Korgaonkar MS, Grieve SM, Galatzer-Levy I, Fox PT, Etkin A (2015) Identification of a common neurobiological substrate for mental illness. JAMA Psychiatry 72:305–315. 25651064 10.1001/jamapsychiatry.2014.2206 PMC4791058

[CIT0059] Goodman WK , Price LH, Rasmussen SA, Delgado PL, Heninger GR, Charney DS (1989a) Efficacy of fluvoxamine in obsessive-compulsive disorder. A double-blind comparison with placebo. Arch Gen Psychiatry 46:36–44. 2491940 10.1001/archpsyc.1989.01810010038006

[CIT0060] Goodman WK , Price LH, Rasmussen SA, Mazure C, Delgado P, Heninger GR, Charney DS (1989b) The Yale-Brown obsessive compulsive scale: II. Validity. Arch Gen Psychiatry 46:1012–1016. 2510699 10.1001/archpsyc.1989.01810110054008

[CIT0061] Goodman WK , Price LH, Rasmussen SA, Mazure C, Fleischmann RL, Hill CL, Heninger GR, Charney DS (1989c) The Yale-Brown obsessive compulsive scale: I. Development, use, and reliability. Arch Gen Psychiatry 46:1006–1011. 2684084 10.1001/archpsyc.1989.01810110048007

[CIT0062] Grandjean J , Buehlmann D, Buerge M, Sigrist H, Seifritz E, Vollenweider FX, Pryce CR, Rudin M (2021) Psilocybin exerts distinct effects on resting state networks associated with serotonin and dopamine in mice. Neuroimage 225:117456. 33069863 10.1016/j.neuroimage.2020.117456

[CIT0063] Grassi G , Cecchelli C, Vignozzi L, Pacini S (2020) Investigational and experimental drugs to treat obsessive-compulsive disorder. J Exp Pharmacol 12:695–706. 33447096 10.2147/JEP.S255375 PMC7801912

[CIT0064] Griffiths RR , Johnson MW, Richards WA, Richards BD, McCann U, Jesse R (2011) Psilocybin occasioned mystical-type experiences: immediate and persisting dose-related effects. Psychopharmacology (Berl) 218:649–665. 21674151 10.1007/s00213-011-2358-5 PMC3308357

[CIT0065] Griffiths RR , Johnson MW, Carducci MA, Umbricht A, Richards WA, Richards BD, Cosimano MP, Klinedinst MA (2016) Psilocybin produces substantial and sustained decreases in depression and anxiety in patients with life-threatening cancer: a randomized double-blind trial. J Psychopharmacol 30:1181–1197. 27909165 10.1177/0269881116675513 PMC5367557

[CIT0066] Grinspoon L , Bakalar JB (1981) The psychedelic drug therapies. Curr Psychiatr Ther 20:275–283. 7326971

[CIT0067] Grob CS , Danforth AL, Chopra GS, Hagerty M, McKay CR, Halberstadt AL, Greer GR (2011) Pilot study of psilocybin treatment for anxiety in patients with advanced-stage cancer. Arch Gen Psychiatry 68:71–78. 20819978 10.1001/archgenpsychiatry.2010.116

[CIT0068] Halberstadt AL , Geyer MA (2011) Multiple receptors contribute to the behavioral effects of indoleamine hallucinogens. Neuropharmacology 61:364–381. 21256140 10.1016/j.neuropharm.2011.01.017 PMC3110631

[CIT0069] Hanes KR (1996) Serotonin, psilocybin, and body dysmorphic disorder: a case report. J Clin Psychopharmacol 16:188–189. 8690834 10.1097/00004714-199604000-00011

[CIT0070] Hartogsohn I (2017) Constructing drug effects: a history of set and setting. Drug Sci Policy Law 3:2050324516683325.

[CIT0071] Hofmann A , Heim R, Brack A, Kobel H, Frey A, Ott H, Petrzilka T, Troxler F (1959) Psilocybin und Psilocin, zwei psychotrope Wirkstoffe aus mexikanischen Rauschpilzen. Helv Chim Acta 42:1557–1572.

[CIT0072] Holze F , Becker AM, Kolaczynska KE, Duthaler U, Liechti ME (2023) Pharmacokinetics and pharmacodynamics of oral psilocybin administration in healthy participants. Clin Pharmacol Ther 113:822–831. 36507738 10.1002/cpt.2821

[CIT0073] Holze F , Erne L, Duthaler U, Liechti ME (2024) Pharmacokinetics, pharmacodynamics and urinary recovery of oral lysergic acid diethylamide administration in healthy participants. Br J Clin Pharmacol 90:200–208. 37596682 10.1111/bcp.15887

[CIT0074] Issari Y , Jakubovski E, Bartley CA, Pittenger C, Bloch MH (2016) Early onset of response with selective serotonin reuptake inhibitors in obsessive-compulsive disorder: a meta-analysis. J Clin Psychiatry 77:e605–e611. 27249090 10.4088/JCP.14r09758

[CIT0075] Jacobs E (2020) A potential role for psilocybin in the treatment of obsessive-compulsive disorder. J Psychedelic Stud 4:77–87.

[CIT0076] Jenike MA (1993) Obsessive-compulsive disorder: efficacy of specific treatments as assessed by controlled trials. Psychopharmacol Bull 29:487–499. 8084980

[CIT0077] Jensen AA , Halberstadt AL, Märcher-Rørsted E, Odland AU, Chatha M, Speth N, Liebscher G, Hansen M, Bräuner-Osborne H, Palner M, Andreasen JT, Kristensen JL (2020) The selective 5-HT2A receptor agonist 25CN-NBOH: structure-activity relationship, in vivo pharmacology, and in vitro and ex vivo binding characteristics of [3H]25CN-NBOH. Biochem Pharmacol 177:113979. 32298690 10.1016/j.bcp.2020.113979

[CIT0078] Johansen P , Krebs TS (2015) Psychedelics not linked to mental health problems or suicidal behavior: a population study. J Psychopharmacol 29:270–279. 25744618 10.1177/0269881114568039

[CIT0079] Johnson M , Richards W, Griffiths R (2008) Human hallucinogen research: guidelines for safety. J Psychopharmacol 22:603–620. 18593734 10.1177/0269881108093587 PMC3056407

[CIT0080] Karno M , Golding JM, Sorenson SB, Burnam MA (1988) The epidemiology of obsessive-compulsive disorder in five US communities. Arch Gen Psychiatry 45:1094–1099. 3264144 10.1001/archpsyc.1988.01800360042006

[CIT0081] Kelly JR , Gillan CM, Prenderville J, Kelly C, Harkin A, Clarke G, O’Keane V (2021) Psychedelic therapy’s transdiagnostic effects: a research domain criteria (RDoC) perspective. Front Psychiatry 12:800072. 34975593 10.3389/fpsyt.2021.800072 PMC8718877

[CIT0082] Kelmendi B , Kichuk SA, DePalmer G, Maloney G, Ching THW, Belser A, Pittenger C (2022) Single-dose psilocybin for treatment-resistant obsessive-compulsive disorder: a case report. Heliyon 8:e12135. 36536916 10.1016/j.heliyon.2022.e12135 PMC9758406

[CIT0083] Kessler RC , Petukhova M, Sampson NA, Zaslavsky AM, Wittchen HU (2012) Twelve-month and lifetime prevalence and lifetime morbid risk of anxiety and mood disorders in the United States. Int J Methods Psychiatr Res 21:169–184. 22865617 10.1002/mpr.1359 PMC4005415

[CIT0084] Khan I , Jaura TA, Tukruna A, Arif A, Tebha SS, Nasir S, Mukherjee D, Masroor N, Yosufi A (2023) Use of selective alternative therapies for treatment of OCD. Neuropsychiatr Dis Treat 19:721–732. 37041856 10.2147/NDT.S403997 PMC10083036

[CIT0085] King AR , Martin IL, Melville KA (1974) Reversal learning enhanced by lysergic acid diethylamide (LSD): concomitant rise in brain 5-hydroxytryptamine levels. Br J Pharmacol 52:419–426. 4458849 10.1111/j.1476-5381.1974.tb08611.x PMC1777004

[CIT0086] Koch K , Reeß TJ, Rus OG, Gürsel DA, Wagner G, Berberich G, Zimmer C (2018) Increased default mode network connectivity in obsessive–compulsive disorder during reward processing. Front Psychiatry 9:254. 29951007 10.3389/fpsyt.2018.00254 PMC6008536

[CIT0087] Krupitsky EM , Grinenko AY (1997) Ketamine psychedelic therapy (KPT): a review of the results of ten years of research. J Psychoactive Drugs 29:165–183. 9250944 10.1080/02791072.1997.10400185

[CIT0088] Kyzar EJ , Stewart AM, Kalueff AV (2016) Effects of LSD on grooming behavior in serotonin transporter heterozygous (Sert⁺/⁻) mice. Behav Brain Res 296:47–52. 26340513 10.1016/j.bbr.2015.08.018

[CIT0089] Leonard HL , Rapoport JL (1987) Relief of obsessive-compulsive symptoms by LSD and psilocin. Am J Psychiatry 144:1239–1240. 10.1176/ajp.144.9.1239b 3631327

[CIT0090] Ley L , Holze F, Arikci D, Becker AM, Straumann I, Klaiber A, Coviello F, Dierbach S, Thomann J, Duthaler U, Luethi D, Varghese N, Eckert A, Liechti ME (2023) Comparative acute effects of mescaline, lysergic acid diethylamide, and psilocybin in a randomized, double-blind, placebo-controlled cross-over study in healthy participants. Neuropsychopharmacology 48:1659–1667. 37231080 10.1038/s41386-023-01607-2 PMC10517157

[CIT0091] Lugo-Radillo A , Cortes-Lopez J (2021) Long-term amelioration of OCD symptoms in a patient with chronic consumption of psilocybin-containing mushrooms. J Psychoactive Drugs 53:146–148. 33225874 10.1080/02791072.2020.1849879

[CIT0092] Ly C , Greb AC, Cameron LP, Wong JM, Barragan EV, Wilson PC, Burbach KF, Soltanzadeh Zarandi S, Sood A, Paddy MR, Duim WC, Dennis MY, McAllister AK, Ori-McKenney KM, Gray JA, Olson DE (2018) Psychedelics promote structural and functional neural plasticity. Cell Rep 23:3170–3182. 29898390 10.1016/j.celrep.2018.05.022 PMC6082376

[CIT0093] Madsen MK , Fisher PM, Burmester D, Dyssegaard A, Stenbæk DS, Kristiansen S, Johansen SS, Lehel S, Linnet K, Svarer C, Erritzoe D, Ozenne B, Knudsen GM (2019) Psychedelic effects of psilocybin correlate with serotonin 2A receptor occupancy and plasma psilocin levels. Neuropsychopharmacology 44:1328–1334. 30685771 10.1038/s41386-019-0324-9 PMC6785028

[CIT0094] Madsen MK , Fisher PM, Stenbæk DS, Kristiansen S, Burmester D, Lehel S, Páleníček T, Kuchař M, Svarer C, Ozenne B, Knudsen GM (2020) A single psilocybin dose is associated with long-term increased mindfulness, preceded by a proportional change in neocortical 5-HT2A receptor binding. Eur Neuropsychopharmacol 33:71–80. 32146028 10.1016/j.euroneuro.2020.02.001

[CIT0095] Majić T , Jungaberle H, Schmidt TT, Zeuch A, Hermle L, Gallinat J (2017) [Psychotherapy with adjuvant use of serotonergic psychoactive substances: possibilities and challenges]. Fortschr Neurol Psychiatr 85:383–392. 28768346 10.1055/s-0043-103085

[CIT0096] Marchesi C , Tonna M, Maggini C (2009) Obsessive-compulsive disorder followed by psychotic episode in long-term ecstasy misuse. World J Biol Psychiatry 10:599–602. 17853269 10.1080/15622970701459828

[CIT0097] Martinotti G , Chiappini S, Pettorruso M, Mosca A, Miuli A, Di Carlo F, D’Andrea G, Collevecchio R, Di Muzio I, Sensi SL, Di Giannantonio M (2021) Therapeutic potentials of ketamine and esketamine in obsessive-compulsive disorder (OCD), substance use disorders (SUD) and eating disorders (ED): a review of the current literature. Brain Sci 11:856. 34199023 10.3390/brainsci11070856 PMC8301752

[CIT0098] Matsushima Y , Shirota O, Kikura-Hanajiri R, Goda Y, Eguchi F (2009) Effects of Psilocybe argentipes on marble-burying behavior in mice. Biosci Biotechnol Biochem 73:1866–1868. 19661714 10.1271/bbb.90095

[CIT0099] McCulloch DE , Madsen MK, Stenbæk DS, Kristiansen S, Ozenne B, Jensen PS, Knudsen GM, Fisher PM (2022) Lasting effects of a single psilocybin dose on resting-state functional connectivity in healthy individuals. J Psychopharmacol 36:74–84. 34189985 10.1177/02698811211026454 PMC8801642

[CIT0100] McDougle CJ , Epperson CN, Pelton GH, Wasylink S, Price LH (2000) A double-blind, placebo-controlled study of risperidone addition in serotonin reuptake inhibitor-refractory obsessive-compulsive disorder. Arch Gen Psychiatry 57:794–801. 10920469 10.1001/archpsyc.57.8.794

[CIT0101] Menzies L , Chamberlain SR, Laird AR, Thelen SM, Sahakian BJ, Bullmore ET (2008) Integrating evidence from neuroimaging and neuropsychological studies of obsessive-compulsive disorder: the orbitofronto-striatal model revisited. Neurosci Biobehav Rev 32:525–549. 18061263 10.1016/j.neubiorev.2007.09.005 PMC2889493

[CIT0102] Modlin NL , Miller TM, Rucker JJ, Kirlic N, Lennard-Jones M, Schlosser D, Aaronson ST (2023) Optimizing outcomes in psilocybin therapy: considerations in participant evaluation and preparation. J Affect Disord 326:18–25. 36707036 10.1016/j.jad.2023.01.077

[CIT0103] Molero P , Ramos-Quiroga JA, Martin-Santos R, Calvo-Sánchez E, Gutiérrez-Rojas L, Meana JJ (2018) Antidepressant efficacy and tolerability of ketamine and esketamine: a critical review. CNS Drugs 32:411–420. 29736744 10.1007/s40263-018-0519-3

[CIT0104] Montgomery SA , Manceaux A (1992) Fluvoxamine in the treatment of obsessive–compulsive disorder. Int Clin Psychopharmacol 7:5–9. 10.1097/00004850-199206001-00003 1355499

[CIT0105] Moreno FA , Delgado PL (1997) Hallucinogen-induced relief of obsessions and compulsions. Am J Psychiatry 154:1037–1038. 10.1176/ajp.154.7.1037b 9210762

[CIT0106] Moreno FA , Wiegand CB, Taitano EK, Delgado PL (2006) Safety, tolerability, and efficacy of psilocybin in 9 patients with obsessive-compulsive disorder. J Clin Psychiatry 67:1735–1740. 17196053 10.4088/jcp.v67n1110

[CIT0107] Moreno JL , Holloway T, Albizu L, Sealfon SC, González-Maeso J (2011) Metabotropic glutamate mGlu2 receptor is necessary for the pharmacological and behavioral effects induced by hallucinogenic 5-HT2A receptor agonists. Neurosci Lett 493:76–79. 21276828 10.1016/j.neulet.2011.01.046 PMC3064746

[CIT0108] Moreton SG , Burden-Hill A, Menzies RE (2023) Reduced death anxiety and obsessive beliefs as mediators of the therapeutic effects of psychedelics on obsessive–compulsive disorder symptomology. Clin Psychol 27:58–73.

[CIT0109] Müller F , Lenz C, Dolder P, Lang U, Schmidt A, Liechti M, Borgwardt S (2017) Increased thalamic resting-state connectivity as a core driver of LSD-induced hallucinations. Acta Psychiatr Scand 136:648–657. 28940312 10.1111/acps.12818 PMC5698745

[CIT0110] Murphy RJ , Godfrey K, Shaw AD, Muthukumaraswamy S, Sumner RL (2024) Modulation of long-term potentiation following microdoses of LSD captured by thalamo-cortical modelling in a randomised, controlled trial. BMC Neurosci 25:7. 38317077 10.1186/s12868-024-00844-5 PMC10845757

[CIT0111] Nasir M , Trujillo D, Levine J, Dwyer JB, Rupp ZW, Bloch MH (2020) Glutamate systems in DSM-5 anxiety disorders: their role and a review of glutamate and GABA psychopharmacology. Front Psychiatry 11:548505. 33329087 10.3389/fpsyt.2020.548505 PMC7710541

[CIT0112] Nichols DE (2004) Hallucinogens. Pharmacol Ther 101:131–181. 14761703 10.1016/j.pharmthera.2003.11.002

[CIT0113] Nichols CD , Sanders-Bush E (2002) A single dose of lysergic acid diethylamide influences gene expression patterns within the mammalian brain. Neuropsychopharmacology 26:634–642. 11927188 10.1016/S0893-133X(01)00405-5

[CIT0114] Niciu MJ , Grunschel BD, Corlett PR, Pittenger C, Bloch MH (2013) Two cases of delayed-onset suicidal ideation, dysphoria and anxiety after ketamine infusion in patients with obsessive-compulsive disorder and a history of major depressive disorder. J Psychopharmacol 27:651–654. 23676198 10.1177/0269881113486718

[CIT0115] Nutt D (2019) Psychedelic drugs-a new era in psychiatry? Dialogues Clin Neurosci 21:139–147. 31636488 10.31887/DCNS.2019.21.2/dnutt PMC6787540

[CIT0116] Odland AU , Kristensen JL, Andreasen JT (2021a) Investigating the role of 5-HT2A and 5-HT2C receptor activation in the effects of psilocybin, DOI, and citalopram on marble burying in mice. Behav Brain Res 401:113093. 33359368 10.1016/j.bbr.2020.113093

[CIT0117] Odland AU , Jessen L, Kristensen JL, Fitzpatrick CM, Andreasen JT (2021b) The 5-hydroxytryptamine 2A receptor agonists DOI and 25CN-NBOH decrease marble burying and reverse 8-OH-DPAT-induced deficit in spontaneous alternation. Neuropharmacology 183:107838. 31693871 10.1016/j.neuropharm.2019.107838

[CIT0118] Passie T , Seifert J, Schneider U, Emrich HM (2002) The pharmacology of psilocybin. Addict Biol 7:357–364. 14578010 10.1080/1355621021000005937

[CIT0119] Passie T , Halpern JH, Stichtenoth DO, Emrich HM, Hintzen A (2008) The pharmacology of lysergic acid diethylamide: a review. CNS Neurosci Ther 14:295–314. 19040555 10.1111/j.1755-5949.2008.00059.x PMC6494066

[CIT0120] Pittenger C (2015) Glutamatergic agents for OCD and related disorders. Curr Treat Options Psychiatry 2:271–283. 26301176 10.1007/s40501-015-0051-8 PMC4540409

[CIT0121] Pittenger C , Bloch MH, Williams K (2011) Glutamate abnormalities in obsessive–compulsive disorder: neurobiology, pathophysiology, and treatment. Pharmacol Ther 132:314–332. 21963369 10.1016/j.pharmthera.2011.09.006 PMC3205262

[CIT0122] Pittenger C , Dulawa S, Thompson SL (2017) ANIMAL MODELS OF OCD. Obsessive-compulsive disorder: phenomenology, pathophysiology, and treatment. New York, NY: Oxford University Press, p 323.

[CIT0123] Pokorny T , Preller KH, Kraehenmann R, Vollenweider FX (2016) Modulatory effect of the 5-HT1A agonist buspirone and the mixed non-hallucinogenic 5-HT1A/2A agonist ergotamine on psilocybin-induced psychedelic experience. Eur Neuropsychopharmacol 26:756–766. 26875114 10.1016/j.euroneuro.2016.01.005

[CIT0124] Pond HL , Heller AT, Gural BM, McKissick OP, Wilkinson MK, Manzini MC (2021) Digging behavior discrimination test to probe burrowing and exploratory digging in male and female mice. J Neurosci Res 99:2046–2058. 34048600 10.1002/jnr.24857 PMC9066774

[CIT0125] Preller KH , Burt JB, Ji JL, Schleifer CH, Adkinson BD, Stämpfli P, Seifritz E, Repovs G, Krystal JH, Murray JD, Vollenweider FX, Anticevic A (2018) Changes in global and thalamic brain connectivity in LSD-induced altered states of consciousness are attributable to the 5-HT2A receptor. eLife 7:e35082. 30355445 10.7554/eLife.35082 PMC6202055

[CIT0126] Preller KH , Razi A, Zeidman P, Stämpfli P, Friston KJ, Vollenweider FX (2019) Effective connectivity changes in LSD-induced altered states of consciousness in humans. Proc Natl Acad Sci U S A 116:2743–2748. 30692255 10.1073/pnas.1815129116 PMC6377471

[CIT0127] Preller KH , Duerler P, Burt JB, Ji JL, Adkinson B, Stämpfli P, Seifritz E, Repovš G, Krystal JH, Murray JD, Anticevic A, Vollenweider FX (2020) Psilocybin induces time-dependent changes in global functional connectivity. Biol Psychiatry 88:197–207. 32111343 10.1016/j.biopsych.2019.12.027

[CIT0128] Roberts MHT , Bradley PB (1967) Studies on the effects of drugs on performance of a delayed discrimination. Physiol Behav 2:389–397.

[CIT0129] Robins LN , Helzer JE, Weissman MM, Orvaschel H, Gruenberg E, Burke JD Jr, Regier DA (1984) Lifetime prevalence of specific psychiatric disorders in three sites. Arch Gen Psychiatry 41:949–958. 6332590 10.1001/archpsyc.1984.01790210031005

[CIT0131] Roseman L , Nutt DJ, Carhart-Harris RL (2018) Quality of acute psychedelic experience predicts therapeutic efficacy of psilocybin for treatment-resistant depression. Front Pharmacol 8:974. 29387009 10.3389/fphar.2017.00974 PMC5776504

[CIT0132] Ross S , Bossis A, Guss J, Agin-Liebes G, Malone T, Cohen B, Mennenga SE, Belser A, Kalliontzi K, Babb J, Su Z, Corby P, Schmidt BL (2016) Rapid and sustained symptom reduction following psilocybin treatment for anxiety and depression in patients with life-threatening cancer: a randomized controlled trial. J Psychopharmacol 30:1165–1180. 27909164 10.1177/0269881116675512 PMC5367551

[CIT0133] Rucker JJ , Jelen LA, Flynn S, Frowde KD, Young AH (2016) Psychedelics in the treatment of unipolar mood disorders: a systematic review. J Psychopharmacol 30:1220–1229. 27856684 10.1177/0269881116679368

[CIT0134] Ruscio AM , Stein DJ, Chiu WT, Kessler RC (2010) The epidemiology of obsessive-compulsive disorder in the National Comorbidity Survey Replication. Mol Psychiatry 15:53–63. 18725912 10.1038/mp.2008.94 PMC2797569

[CIT0135] Savage C , Savage E, Fadiman J, Harman W (1964) LSD: therapeutic effects of the psychedelic experience. Psychol Rep 14:111–120.

[CIT0136] Saxena S , Wang D, Bystritsky A, Baxter LR Jr (1996) Risperidone augmentation of SRI treatment for refractory obsessive-compulsive disorder. J Clin Psychiatry 57:303–306. 8666572

[CIT0137] Shao L-X , Liao C, Gregg I, Davoudian PA, Savalia NK, Delagarza K, Kwan AC (2021) Psilocybin induces rapid and persistent growth of dendritic spines in frontal cortex in vivo. Neuron 109:2535–2544.e4. 34228959 10.1016/j.neuron.2021.06.008 PMC8376772

[CIT0138] Singh S , Botvinnik A, Shahar O, Wolf G, Yakobi C, Saban M, Salama A, Lotan A, Lerer B, Lifschytz T (2023) Effect of psilocybin on marble burying in ICR mice: role of 5-HT1A receptors and implications for the treatment of obsessive-compulsive disorder. Transl Psychiatry 13:164. 37164956 10.1038/s41398-023-02456-9 PMC10172379

[CIT0139] Singleton SP , Luppi AI, Carhart-Harris RL, Cruzat J, Roseman L, Nutt DJ, Deco G, Kringelbach ML, Stamatakis EA, Kuceyeski A (2022) Receptor-informed network control theory links LSD and psilocybin to a flattening of the brain’s control energy landscape. Nat Commun 13:5812. 36192411 10.1038/s41467-022-33578-1 PMC9530221

[CIT0140] Soto D , Theodoraki M, Paz-Alonso PM (2018) How the human brain introspects about one’s own episodes of cognitive control. Cortex 107:110–120. 29198443 10.1016/j.cortex.2017.10.016

[CIT0141] Sousa TR , Rema J, Machado S, Novais F (2022) Psychedelics and hallucinogens in psychiatry: finding new pharmacological targets. Curr Top Med Chem 22:1250–1260. 34852736 10.2174/1568026621666211201145800

[CIT0142] Stein DJ , Costa DLC, Lochner C, Miguel EC, Reddy YCJ, Shavitt RG, van den Heuvel OA, Simpson HB (2019) Obsessive-compulsive disorder. Nat Rev Dis Primers 5:52. 31371720 10.1038/s41572-019-0102-3 PMC7370844

[CIT0143] Studerus E , Kometer M, Hasler F, Vollenweider FX (2011) Acute, subacute and long-term subjective effects of psilocybin in healthy humans: a pooled analysis of experimental studies. J Psychopharmacol 25:1434–1452. 20855349 10.1177/0269881110382466

[CIT0144] Swierkosz-Lenart K , Dos Santos JFA, Elowe J, Clair AH, Bally JF, Riquier F, Bloch J, Draganski B, Clerc MT, Pozuelo Moyano B, von Gunten A, Mallet L (2023) Therapies for obsessive-compulsive disorder: current state of the art and perspectives for approaching treatment-resistant patients. Front Psychiatry 14:1065812. 36873207 10.3389/fpsyt.2023.1065812 PMC9978117

[CIT0145] Tang W , Zhu Q, Gong X, Zhu C, Wang Y, Chen S (2016) Cortico-striato-thalamo-cortical circuit abnormalities in obsessive-compulsive disorder: a voxel-based morphometric and fMRI study of the whole brain. Behav Brain Res 313:17–22. 27388149 10.1016/j.bbr.2016.07.004

[CIT0146] Taylor G (2017) Marble burying as compulsive behaviors in male and female mice. Acta Neurobiol Exp 77:254–260. 29182616

[CIT0147] Taylor S , Asmundson GJ, Jang KL (2016) Etiology of obsessions and compulsions: General and specific genetic and environmental factors. Psychiatry Res 237:17–21. 26921046 10.1016/j.psychres.2016.01.071

[CIT0148] Thomas A , Burant A, Bui N, Graham D, Yuva-Paylor LA, Paylor R (2009) Marble burying reflects a repetitive and perseverative behavior more than novelty-induced anxiety. Psychopharmacology (Berl) 204:361–373. 19189082 10.1007/s00213-009-1466-y PMC2899706

[CIT0149] Thompson S , Dulawa S (2017) Pharmacological and behavioral rodent models of OCD. Obsessive-compulsive disorder: phenomenology, pathophysiology, and treatment. New York, NY: Oxford University Press, pp 385–400.

[CIT0150] Tiwari P , Ehrenkranz R, Bryce Yaden D (2024) Psychiatric applications of psychedelics: neurobiological foundations for treatments of depression, anxiety, and obsessive-compulsive disorder. Adv Psychiatry Behav Health 4:47–56.

[CIT0151] Vaidya VA , Marek GJ, Aghajanian GK, Duman RS (1997) 5-HT2A receptor-mediated regulation of brain-derived neurotrophic factor mRNA in the hippocampus and the neocortex. J Neurosci 17:2785–2795. 9092600 10.1523/JNEUROSCI.17-08-02785.1997 PMC6573109

[CIT0152] van Amsterdam J , van den Brink W (2022) The therapeutic potential of psilocybin: a systematic review. Expert Opin Drug Saf 21:833–840. 35225143 10.1080/14740338.2022.2047929

[CIT0153] Van der Straten A , Denys D, Van Wingen G (2017) Impact of treatment on resting cerebral blood flow and metabolism in obsessive–compulsive disorder: a meta-analysis. Sci Rep 7:17464. 29234089 10.1038/s41598-017-17593-7 PMC5727319

[CIT0154] Van Schalkwyk GI , Bhalla IP, Griepp M, Kelmendi B, Davidson L, Pittenger C (2016) Toward understanding the heterogeneity in obsessive-compulsive disorder: Evidence from narratives in adult patients. Aust N Z J Psychiatry 50:74–81. 25855685 10.1177/0004867415579919 PMC4598276

[CIT0155] Vollenweider FX , Vollenweider-Scherpenhuyzen MF, Bäbler A, Vogel H, Hell D (1998) Psilocybin induces schizophrenia-like psychosis in humans via a serotonin-2 agonist action. Neuroreport 9:3897–3902. 9875725 10.1097/00001756-199812010-00024

[CIT0156] Walker ER , McGee RE, Druss BG (2015) Mortality in mental disorders and global disease burden implications: a systematic review and meta-analysis. JAMA Psychiatry 72:334–341. 25671328 10.1001/jamapsychiatry.2014.2502 PMC4461039

[CIT0157] Wilcox JA (2014) Psilocybin and obsessive–compulsive disorder. J Psychoactive Drugs 46:393–395. 25364991 10.1080/02791072.2014.963754

[CIT0158] Zambrano-Vazquez L , Allen JJB (2014) Differential contributions of worry, anxiety, and obsessive–compulsive symptoms to ERN amplitudes in response monitoring and reinforcement learning tasks. Neuropsychologia 61:197–209. 24971709 10.1016/j.neuropsychologia.2014.06.023

[CIT0159] Zghoul T , Blier P (2003) Enhancing action of LSD on neuronal responsiveness to serotonin in a brain structure involved in obsessive-compulsive disorder. Int J Neuropsychopharmacol 6:13–21. 12899732 10.1017/S1461145702003218

